# Photothermal heating of titanium nitride nanomaterials for fast and uniform laser warming of cryopreserved biomaterials

**DOI:** 10.3389/fbioe.2022.957481

**Published:** 2022-08-25

**Authors:** Crysthal Alvarez, Carla Berrospe-Rodriguez, Chaolumen Wu, Jacqueline Pasek-Allen, Kanav Khosla, John Bischof, Lorenzo Mangolini, Guillermo Aguilar

**Affiliations:** ^1^ J. Mike Walker ’66 Department of Mechanical Engineering, Texas A&M University, College Station, TX, United States; ^2^ Department of Mechanical Engineering, University of California, Riverside, Riverside, CA, United States; ^3^ Department of Chemistry, University of California, Riverside, Riverside, CA, United States; ^4^ Department of Biomedical Engineering, University of Minnesota, Minneapolis, MN, United States; ^5^ Department of Mechanical Engineering, University of Minnesota, Minneapolis, MN, United States

**Keywords:** titanium nitride (TiN), nanoparticles, clusters, nanowarming, cryopreservation, biomaterials, plasmonics

## Abstract

Titanium nitride (TiN) is presented as an alternative plasmonic nanomaterial to the commonly used gold (Au) for its potential use in laser rewarming of cryopreserved biomaterials. The rewarming of vitrified, glass like state, cryopreserved biomaterials is a delicate process as potential ice formation leads to mechanical stress and cracking on a macroscale, and damage to cell walls and DNA on a microscale, ultimately leading to the destruction of the biomaterial. The use of plasmonic nanomaterials dispersed in cryoprotective agent solutions to rapidly convert optical radiation into heat, generally supplied by a focused laser beam, proposes a novel approach to overcome this difficulty. This study focuses on the performance of TiN nanoparticles (NPs), since they present high thermal stability and are inexpensive compared to Au. To uniformly warm up the nanomaterial solutions, a beam splitting laser system was developed to heat samples from multiple sides with equal beam energy distribution. In addition, uniform laser warming requires equal distribution of absorption and scattering properties in the nanomaterials. Preliminary results demonstrated higher absorption but less scattering in TiN NPs than Au nanorods (GNRs). This led to the development of TiN clusters, synthetized by nanoparticle agglomeration, to increase the scattering cross-section of the material. Overall, this study analyzed the heating rate, thermal efficiency, and heating uniformity of TiN NPs and clusters in comparison to GNRs at different solution concentrations. TiN NPs and clusters demonstrated higher heating rates and solution temperatures, while only clusters led to a significantly improved uniformity in heating. These results highlight a promising alternative plasmonic nanomaterial to rewarm cryopreserved biological systems in the future.

## 1 Introduction

Cryopreservation is an important method to preserve a range of biological systems for various applications, including for biodiversity preservation (Jang et al., 2017). This thermal process preserves living cells (Meryman, 2007), plants (Benson, 1999), germplasm of animals (Mazur, Leibo and Seidel, 2008), and transplantable organs (Finger and Bischof, 2018) to mention some. Cooling down biological samples to a cryogenic temperature range (sub—140°C), cryopreserves them without damaging ice formation to the glass like vitrified state. Ice formation occurs in a two-stage nucleation, then grows during cooling, known as freezing, or upon warming, known as devitrification. Ice is mitigated by cryoprotective agents (CPAs), aqueous solutions of organic solvents (methanol, propylene glycol, dimethyl sulfoxide), salts (osmotic balance), and sugars (nutrients). CPAs protect vital structures by decreasing the rate of cooling or warming to prevent the formation of ice (Rajan and Matsumura, 2018; Chang and Zhao, 2021). Preserving samples at low temperatures allows for storage over prolonged periods of time, due to negligible metabolic processes functioning at these temperature ranges. Also, it allows to store multiple samples of one biological system creating a stock with ease of access, as seen in the fields of cell research (Yokoyama, Thompson and Ehrhardt, 2012).

Biological samples, ranging from droplets to organs, have successfully cryopreserved in a vitrified state maintaining their functional properties intact (Fahy et al., 1984; Daly et al., 2018; Khosla et al., 2020). This process needs CPAs at different concentrations, 0%–50% w/w, depending on the size of the sample, to assist with the cooling process which ranges from few thousands to few hundreds °C/min, respectively (Han and Bischof, 2020). The concentration of CPAs correlates to the critical cooling rate (CCR), needed to avoid ice formation, and the critical warming rate (CWR). The CWR ranges from 10^1^ to 10^12^°C/min, which roughly correlates to a 10^1^–10^4^°C/min CCR, for biological samples ranging in size from organs (L) to droplets (μL), respectively (Han and Bischof, 2020). This study extrapolated data from higher CPA measurements. Recently Kangas et al. (2022), measured the CWR via laser calorimetry in microliter (μL) droplets of low CPA concentrations, 20 to 40 wt%. This study found that the CWR and CCR range from 0.4 × 10^5^–10^7^°C/min and ∼10^4^–10^5^°C/min, respectively. With higher CPA concentrations this process needs lower cooling rates, but the toxicity increases to different degrees (Fahy et al., 1984; Rall and Fahy, 1985; Martino, Songsasen and Leibo, 1996; Hagedorn et al., 1998; Khosla et al., 2017, 2018; Han and Bischof, 2020), depending on the biological samples (Clark, Fahy and Karow, 1984; de Vries et al., 2019). For convective cooling, such as submerging in liquid nitrogen (LN_2_), decreasing the total volume and thickness increases the CCR, requiring less CPA and resulting in less toxicity. Rewarming of small volumes remains a challenge as vitrification at low CPA concentrations is possible (Khosla et al., 2018). The rate and uniformity of rewarming from the cryogenic state results in viability, physical and functional, of the biological system.

Successfully rewarming a cryopreserved biological system requires warming rates orders of magnitude above the cooling rate, without temperature gradients. For example, mouse oocytes have vitrified and successfully rewarmed at rates ranging from 95 to 70,000°C/min and 610 to 118,000°C/min, respectively (Mazur and Seki, 2011). However, rewarming at high rates in the μL scale with low CPA concentrations benefit from the use of plasmonic nanomaterials, which absorb energy from a laser source and distribute it uniformly to avoid crystallization (Khosla et al., 2018; Han and Bischof, 2020). For example, Gold (Au) plasmonic nanoparticles (NPs) rewarmed vitrified biomaterial droplets and achieved warming rates up to 10,000,000°C/min, using a 1,064 nm pulsed laser in the millisecond (*ms*) range, with an average energy of 3 J (Khosla et al., 2018, 2020; Liu, 2020).

The absorption and scattering properties of a plasmonic nanomaterial serve as a crucial factor in achieving high and uniform heating rates. Tunable fabrication of Au nanomaterials facilitates the manipulation of scattering to absorption ratio, which makes them leading materials and widely used in this field of laser nanowarming (Qin and Bischof, 2012; Qin et al., 2016; Liu, 2020). For example, gold nanorods (GNRs) offer superior absorption and scattering properties at small sizes, and more specifically, in the near infrared (NIR) window (Jain et al., 2006). However, the use of Au nanomaterials requires fabrication of complex shapes like rods or shells (Oldenburg et al., 1999; Tsai et al., 2013), in order to achieve absorption in the NIR light for biological applications. Furthermore, Au NPs present poor thermal stability compared to other plasmonic materials and the complex shapes needed to absorb NIR light sometimes experience overheating and reshaping (Puértolas et al., 2015; Wei et al., 2015; Cavigli et al., 2021). This disadvantage correlates to the relatively low melting point of Au (1,000°C), which significantly reduces when nanostructured into complex shapes as a consequence of melting point depression (Jiang, Zhang and Zhao, 2003). Additionally, GNRs often require enhancement of their absorption coefficient in order to improve their photothermal efficiency (Sivapalan et al., 2012, 2013). This is due to their high scattering cross-section, compared to other materials (Ni et al., 2008; He et al., 2010), having an impact on the absorption capacity of GNRs solutions, such as in CPA solutions.

In the recent years, titanium nitride (TiN) emerged as a plasmonic material with good refractory and metallic properties. Several investigations, focused on the optical properties of sputtered TiN thin films, confirmed that this material supports localized surface plasmon resonance in the visible and NIR part of the spectrum (Naik et al., 2012; Naik, Shalaev and Boltasseva, 2013; Li et al., 2014). Most recently, TiN successfully produced in nanoparticle form, thus extending its range of applicability to applications currently limited to gold and silver (Alvarez Barragan et al., 2017; Berrospe Rodriguez et al., 2020). In particular, Alvarez Barragan et al. (2017) combined optical characterization and ab-initio modeling to conclusively confirm the plasmonic nature of the NIR absorption band in TiN NPs. Furthermore, TiN NPs present a thermal stability up to 900°C (Alvarez Barragan et al., 2019; Berrospe Rodriguez et al., 2020).

This study examined TiN as an alternative to Au as it is inexpensive, more reliable, and a simpler plasmonic nanomaterial to achieve rapid and uniform photothermal warming for cryopreservation applications in the future. A comparative study between TiN NPs and GNRs as photothermal agents was performed by means of a thermometry method. A continuous wave (CW) laser beam, divided into multiple beams with the same laser energy, heated solutions at different concentrations. A multiple thermocouple setup, distributed equally within the plasmonic nanoparticle solutions, obtained the heating rate and heat distribution along different solution volumes. These results are discussed through the comparison of the absorption and scattering properties between GNRs and TiN NPs. Furthermore, TiN NPs were assembled into spherical clusters by using a reverse emulsion method (Liu et al., 2015) to increase its scattering cross-section and achieve a uniform heat distribution on the solution to enhance the photothermal efficiency (Liu, 2020). Finally, as a proof of concept, a 1 μL CPA-TiN NPs droplet successfully laser nanowarmed with no evidence of devitrification or cracking.

## 2 Experimental Details

### 2.1 Synthesis and characterization of nanomaterials

**FIGURE 1:**
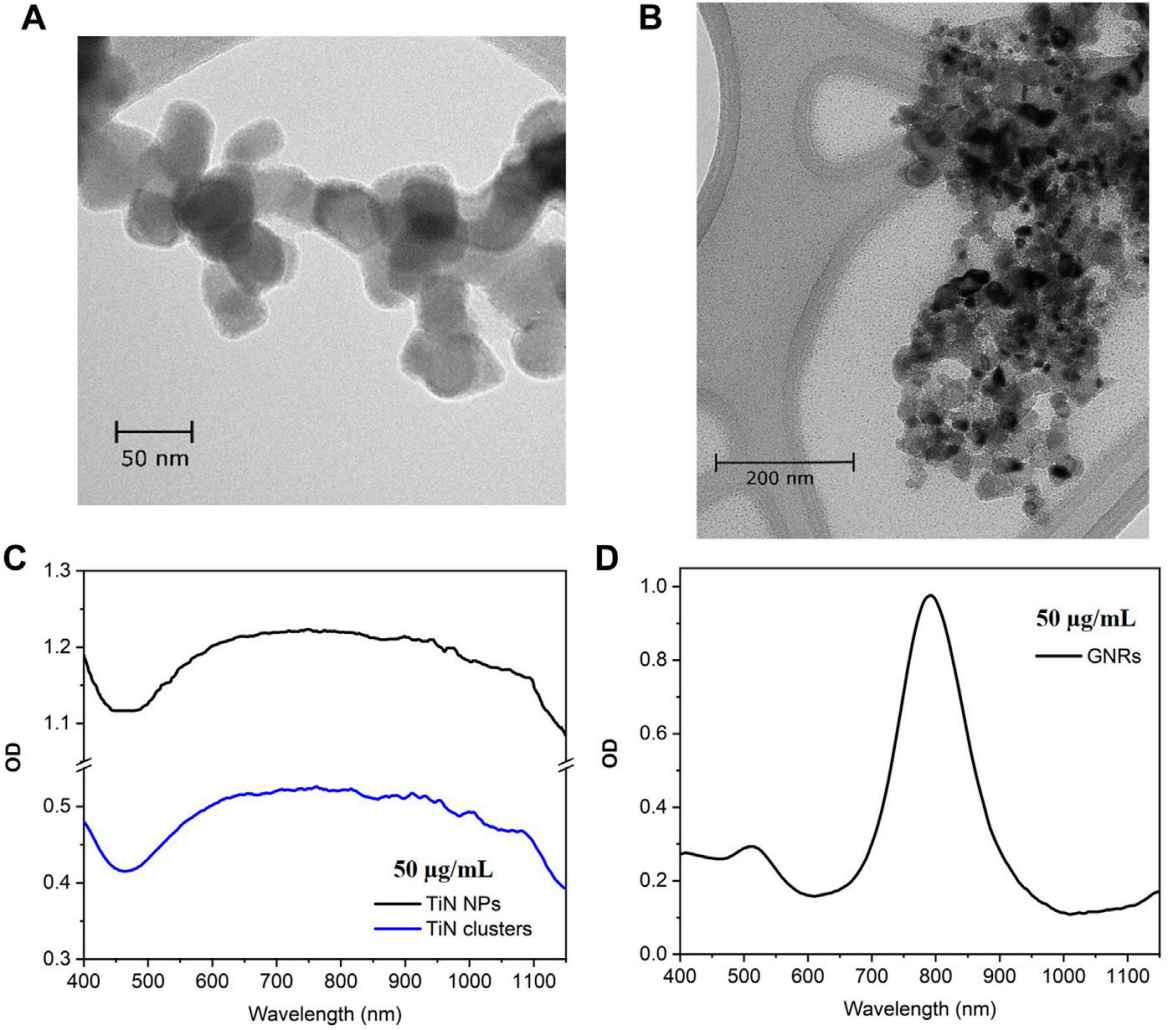
TEM micrographs and optical density (OD). **(A)** Commercial TiN NPs with cubic crystalline structure and average particle size of 50 nm. **(B)** TiN clusters from NPs agglomeration using reverse emulsion method, with average particle size of 300 nm. **(C)** OD spectrum of TiN NPs and TiN clusters centered at λ= 800 nm. **(D)** OD spectrum of GNRs with a plasmonic peak at λ= 808 nm.

The TiN NPs used in this study were purchased from U.S. Research Nanomaterials Inc. with a high purity of 99.2%, a cubic crystalline structure, and an average particle size of 50 nm (see Figure 1A). The NPs show a clear plasmonic resonance in the NIR region with a plasmonic peak at 800 nm.

The TiN clusters were synthetized from the commercial TiN NPs using a reverse emulsion method (Liu et al., 2015, 2021). In a typical assembly, 0.5 mL of TiN NPs solution (20 mg/mL) was added into 5 mL of n-butanol solvent. The mixture was emulsified by sonication for 30 s to achieve agglomeration of particles. The obtained clusters were washed with ethanol once and redispersed in 5 mL of ethanol. This resulted in clusters with the particle distribution size of 300 nm, as demonstrated in the Figure 1B SEM micrograph. Figure 1C shows the OD spectrum for TiN NPs and TiN clusters. Both spectra are centered at λ = 800 nm and there is no shift in the plasmonic peak of the clusters due to nanoparticle agglomeration. However, the OD is 0.5 for TiN clusters, which decreased 50% compared to TiN NPs.

The GNRs (A12-25-808-CTAB) used in this study were purchased from Nanopartz, with an OD of 1 at the plasmonic peak (808 nm) for a concentration of 50 μg/mL (see Figure 1D). The dimensions of the rods are 25 nm in diameter and 90 nm in length. These dimensions make a fair comparison to the TiN NPs presented in this study and to the GNRs already investigated for laser induced warming in Zebrafish embryos (Khosla et al., 2017, Khosla et al.,2020). The TiN NPs and GNRs are in the same magnitude of volume. Additionally, the extinction cross-section of these GNRs matches the laser excitation of our laser and the nanorod effective radius, ∼ 22.7 nm, provides an increase in the ratio of scattering to absorption, which uniform warming requires (Jain et al., 2006; Liu et al., 2020).

### 2.2 Photothermal thermometry setup

**FIGURE 2:**
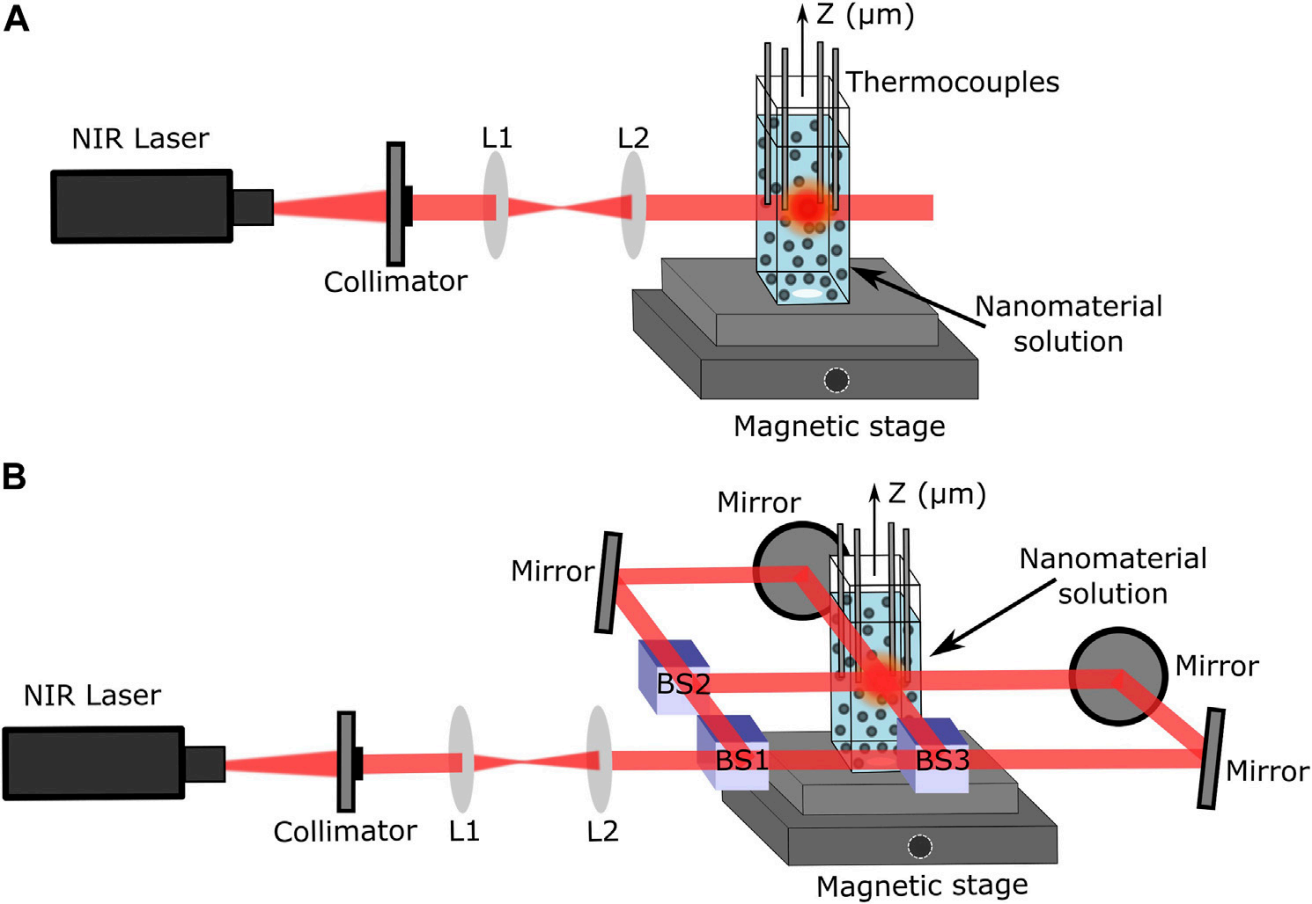
Laser setups using CrystaLaser 808 nm laser. **(A)** Four thermocouple setup for thermal measurements with only 1LB. **(B)** Beam splitting laser setup to uniformly heat solutions from four directions (4LB).

The setups used to perform photothermal thermometry measurements using one laser beam (1LB) and four laser beams (4LB) are shown in Figures 2A,B, respectively. In both cases, a z-micro stage displaced a mounted set of 4 K-type thermocouples, connected to a thermometer reader (Perfect Prime data logger), along a quartz cuvette containing the plasmonic solutions. For both setups, a set of lenses obtained an initial collimated beam with a spot size of approximately 2 mm. Particularly, for the 4LB system, a set of mirrors and 50% beam splitters divided the laser beam into 4 beams, all with same laser energy and spot size, as presented in Figure 2B. Further details of the photothermal thermometry setup are presented in section S.1 of the Supplementary Material.

The solutions were cooled down to room temperature before starting any measurement. The thermocouples, placed equally distant (2 mm) from the laser heating zone, obtained an average temperature of the solution. Both setups used a CW laser at λ = 808 nm (single-mode *CrystaLaser*) to match the plasmonic absorption peak of all the materials, located in the first NIR biological window (see Figure 2).

### 2.3 Stability

Laser rewarming requires colloidal stability of plasmonic NPs in CPAs. Colloidal stability describes the preservation of different properties of the nanocomposites, such as aggregation, composition, optical, surface chemistry, crystallinity, shape, etc. The preservation of the optical properties was the focus due to its direct impact on the thermal performance of the solution. The extinction coefficient of TiN NPs, TiN clusters, and GNRs was evaluated by suspending the nanomaterials in 15% methanol (MeOH), 7.5% propylene glycol (PG), and 20% polyethylene glycol (PEG), a CPA solution developed by Smith et al. (2020) to successfully vitrify a biomaterial microdroplet. Solutions of 5 mL CPAs with concentrations as high as 100 μg/mL of each nanomaterial were sonicated for 6 min to obtain homogenized samples. After transferring the solutions to a quartz cuvette, the transmission spectrum was measured. The extinction coefficient spectrum was calculated from the transmission measurements using the CPA solution without any nanocomposite as a baseline. This procedure was followed for a period of 30 days to evaluate the stability of the material over time. Additionally, the colloidal stability was evaluated by re-sonicating solutions up to 6 times and measuring the extinction coefficient after each sonication.

### 2.4 Cell studies

A preliminary toxicity study of TiN NPs and clusters was performed on Human Dermal Fibroblasts (HDF) cells. These cells are widely used in tissue engineering (Syedain, Weinberg and Tranquillo, 2008; Deng et al., 2009; Sommar et al., 2010; Chandrasekaran et al., 2011) and in toxicity studies of nanomaterials that come in first contact with the skin in topically applied antiseptics (Pernodet et al., 2006; Mateo et al., 2015; Avalos et al., 2016; Galandáková et al., 2016). In addition, HDF cells are widely used in the cryobiology field (Balasubramanian, Bischof and Hubel, 2006; Balasubramanian, Wolkers and Bischof, 2009; Choi and Bischof, 2011, Choi and Bischof, 2013). Despite the increasing research of TiN nanomaterials for biotechnology applications, no toxicity study of TiN NPs on HDF cells exists and current literature regarding their toxicity is very limited (He et al., 2017; Kim et al., 2019; Popov et al., 2019; Nirwan et al., 2021).

The HDF cells were cultured in 96 well plates prior to adding TiN NPs and clusters. Five different concentrations ranging from 12 to 400 μg/mL of both nanomaterials were prepared in cell media. A volume of 200 μL of each concentration was added to a well plate, with a repetition of four to six times of each concentration. For the control sample, only cell media was added to the cells. All well plates were incubated for 24 h and then washed with Phosphate Buffered Saline (PBS) to remove the NPs and clusters. A Hoechst/PI dye was added to all cells in a duration of 15 min for fluorescence imaging purposes (Ciancio et al., 1988). The dye was then removed and PBS was added to image all the HDF cells using an inverted microscope (Olympus IX51).

### 2.5 Microdroplet laser nanowarming

After the thermal characterization of TiN nanomaterials for its potential use on laser nanowarming, a preliminary microdroplet study using plasmonic TiN NPs and CPA solution was performed. The microdroplet consisted of a CPA solution with 100 μg/mL concentration of TiN NPs. The CPA solution, proven as the most efficient for cryopreservation on microscale volumes, consisted of a mixture of 15% MeOH, 7.5% PG, and 20% PEG (Smith et al., 2020). The solution was sonicated and then, using a precision pipette, 1 μL was placed on a cryotop under the pulsed laser rewarming system designed by Khosla et al. (2018) for its use on GNRs nanowarming. In this setup, the microdroplet is mechanically submerged into a LN_2_ container and after vitrifying, it is brough up into the focus of the laser beam. The laser spot consisted of a top hat shape with a diameter of 2 mm and an input laser energy of 3 J.

## 3 Results and Discussion

### 3.1 Heating rate characterization

**FIGURE 3:**
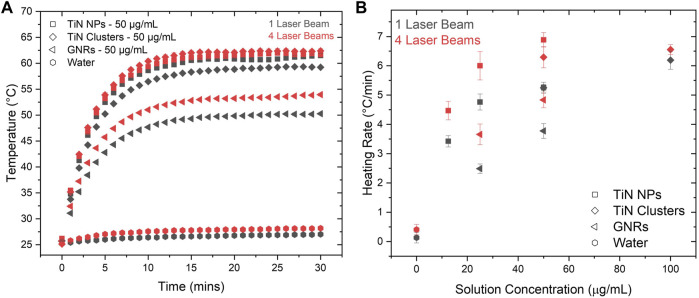
Heating rate comparison between laser setups. **(A)** Temperature increments as a function of time in TiN NPs, TiN clusters, and GNRs at 50 μg/mL concentrations with a volume of 500 μL. This graph shows the difference in photothermal heating for 1LB and 4LB setups. **(B)** The heating rate of TiN NPs, TiN clusters, and GNRs at various concentrations showing the difference between 1LB and 4LB.

For a comparison, both setups, 1LB and 4LB (Figures 2A,B, respectively), heated the nanomaterials dissolved in water solutions of 500 μL with different concentrations. The temperature was measured every minute for a duration of 30 min upon reaching stability. Figure 3A shows the temperature increment for solutions with a concentration of 50 μg/mL of all the studied materials. The results demonstrate around 28% higher temperatures for TiN NPs and clusters compared to GNRs, for both heating conditions (1LB and 4LB). TiN NPs have an OD 20% higher than GNRs (OD = 1), therefore, higher absorption leads to higher temperatures. However, despite an OD of 0.5, TiN clusters reach similar temperatures as TiN NPs. This feature attributes to the improvement on the scattering properties of TiN clusters compared to NPs, where the redistribution of photon penetration from the scattered light, reabsorption, leads to a superior photothermal conversion (Roper et al., 2007; Richardson et al., 2009; Liu et al., 2020). Previous work reported the over-estimation of conversion efficiency up to 30% on larger GNRs, when reabsorption was neglected (Liu et al., 2020). According to the results demonstrated in Figure 3A, this reabsorption process seems more efficient on TiN clusters compared to GNRs. However, further investigations were performed before conclusion.

Figure 3B shows the heating rate of 500 μL solutions for TiN NPs, TiN clusters, and GNRs, as a function of particle concentration. This was obtained by fitting the linear increment of temperature before reaching the plateau state (see Figure 3A). For all the cases, the heating rate increases significantly going from the 1LB to the 4LB system, where the energy is equally distributed. This increased the efficiency of the heating process. As mentioned previously, when the biological sample decreases in size, higher cooling and heating rates are required. In few microliter volumes, studies reported heating rates up to 10^6^°C/min using a single *ms* pulsed laser (λ = 1,064 nm) beam in GNRs solutions (Jin and Mazur, 2015; Khosla et al., 2017, 2018, 2020; Daly et al., 2018). Similarly, the distribution of the high energy laser in multiple beams proposes a significant increase in the heating rate in the *ms* laser regime, making the rewarming of biological samples around 1 μL possible.

Looking into the heating rate from the perspective of the material, TiN NPs presented the highest rate up to 7.5°C/min for 50 μg/mL. Following, TiN clusters presented a heating rate of 6.5°C/min for 100 μg/mL and at the end GNRs presented a 5°C/min rate for 50 μg/mL. The TiN NPs, with only ¼ of the concentration (12.5 μg/mL) of GNRs, achieved a similar heating rate. The use of lower particle concentrations to achieve similar heating rates opens the possibility to laser rewarm cryopreserved microorganism with lower levels of toxicity. This potentially increases viability of the microorganisms, which so far results reported only 22% post-laser rewarming (3 h) for Zebrafish embryos (Khosla et al., 2020). Overall, this result demonstrates the advantage of TiN NPs over GNRs to obtain faster heating rates and avoid ice formation during the rewarming of cryopreserved biomaterials (Fowler and Toner, 2005; Khosla et al., 2017).

**FIGURE 4:**
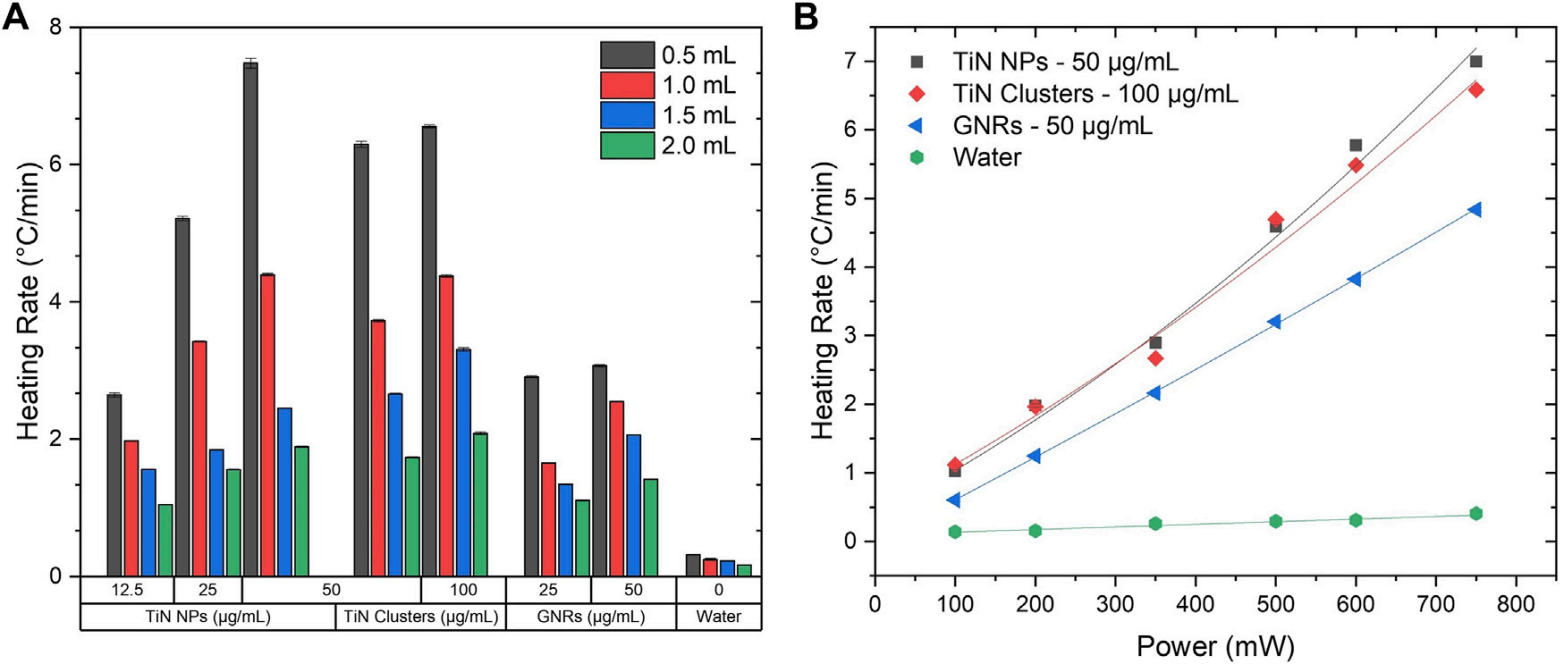
Heating rate maps. **(A)** Heating rate as a function of volume at a maximum laser power of 750 mW using the 4LB setup for various concentrations of TiN NPs, TiN clusters, and GNRs. **(B)** Heating rate as a function of power at 500 μL using the 4LB setup for TiN NPs (50 μg/mL), TiN clusters (100 μg/mL), and GNRs (50 μg/mL).

One of the main goals of laser nanowarming with plasmonic NPs is to cryopreserve small biomaterials (<2 mm), which lowers concentrations of cryoprotectants (2–3 M), using rapid cooling and warming approaches. For example, Bovine oocytes (Martino et al., 1996), Mouse oocytes (Mazur and Seki, 2011), and Zebrafish embryos (Khosla et al., 2017) to mention a few. Therefore, understanding the heating performance of nanomaterials to balance the CCR and the CWR at these volume ranges is crucial. This experiment was performed using the 4LB system and a constant total laser power of 750 mW on the plasmonic solutions from 2 mL to 500 μL. Experiments below volumes of 500 μL were not possible to perform due to the nature of the thermometry setup. However, this measurement provided a good platform for comparing how different plasmonic materials performed under laser-induced heating. Figure 4A shows the heating rate map as a function of volume and particle concentrations for TiN NPs, TiN clusters, and GNRs. Regardless of the material, a significant increase in heating rate is observed as solution volume reduces. However, this is more evident for TiN NPs and clusters, with a maximum heating rate change of Δ = 5.7°C/min at 50 μg/mL and Δ = 4.5°C/min at 100 μg/mL, from to 2 mL to 500 μL, respectively. GNRs only had a rate change of Δ = 2°C/min at 50 μg/mL for the same volume change. Importantly, TiN NPs and clusters reached higher heating rates compared to GNRs at all volumes at the same concentration (50 μg/mL). Additionally, for concentrations of TiN clusters at 50 μg/mL or above, the heating rate approached an exponential-like behavior with volumes similar to TiN NPs, although to a lesser degree. This possibly attributes to the increment of scattering due to particle size increment from NPs to clusters. In contrast, GNRs displayed a linear behavior. The heating rate for TiN clusters falls between the ones obtained for TiN NPs and GNRs. This observation suggests that the clusters absorb more energy from the laser source compared to GNRs, but scatter more light than TiN NPs. To assess this hypothesis, the multiangle light scattering (MALS) technique was used to measure the scattering cross-section of all nanomaterials, as demonstrated below (Section 3.2). The exponential like response of TiN solutions with change in volume suggests that these plasmonic nanomaterials can potentially achieve much higher heating rates compared to GNRs at the microdroplet level (Han and Bischof, 2020). However, further studies require experiments in the microdroplet regime.

Plasmonic solutions propose to rewarm various cryopreserved biomaterials that require different heating rates according to their dimensions and absorption properties. Therefore, it is important to understand the heating response of these solutions in relation to the NIR laser power. At a constant volume of 500 μL, the heating rate of the plasmonic solutions with the highest concentration for each material (50 μg/mL TiN NPs, 100 μg/mL TiN clusters, and 50 μg/mL GNRs) was measured as a function of laser power. Figure 4B shows the best fit to each nanomaterial heating rate with power laser increment. GNRs presented a clear linear behavior from a range of 100–750 mW, whereas TiN nanomaterials best fitted with aP_laser_ + bP_laser_^2^ in the same range. There is a small but visible nonlinear dependency of the laser power with temperature, especially for TiN NPs. This possibly relates to the higher nonlinear absorption of TiN NPs compared to GNRs, previously obtained in our group by Z-scan method, at similar particle concentrations. The study found the nonlinear absorption coefficient of TiN NPs being one order of magnitude higher than GNRs (Sabzeghabae et al., 2021). Therefore, as the absorption of the material increases with the laser power, the heating rate of the TiN NPs and TiN clusters solutions increases as well. This outcome reiterates the potential of TiN achieving higher heating rates compared to GNRs. Importantly, studies have demonstrated TiN NPs being more thermally stable under pulsed laser radiation in comparison to GNRs (Alvarez Barragan et al., 2017, 2019; Sabzeghabae et al., 2021).

### 3.2 Scattering and uniformity

TiN NPs present promising results as an alternative to Au nanomaterials in photothermal processes. Particularly, for its application on laser rewarming of cryopreserved biomaterials. This study demonstrated that TiN nanomaterials achieve higher heating rates under the same light conditions due to their superior absorption. In addition to fast heating, laser rewarming requires a uniform thermal profile. Distribution of the laser energy in the solution dictates this uniformity and it depends on the scattering of the material to convert it into heat. For this reason, the scattering cross-section was calculated for each material using MALS technique (Hulst and van de Hulst, 1981; Cox, DeWeerd and Linden, 2002; Kreibig and Vollmer, 2013; Ogendal, 2016), as described in detail in the Supplementary Material document, section S.2. The scattering intensity for low concentrations of TiN NPs, TiN clusters, and GNRs at 12.5, 25, and 25 μg/mL, respectively, was measured from 0 to 90 degrees in increments of 5 degrees (see Supplementary Figure S2). The scattering cross-section was obtained by Eq. 1:

$$\sigma =\frac{V_{Particle}}{V_{Spot}}\frac{f_{f}^{2}}{I_{O,\;Water}}\int_{0}^{90}I_{\theta }sin\theta \;d\theta$$ (1)

This equation presents $V_{Particle}$ and $V_{Spot}$ as the volume of the nanomaterial and the laser beam spot, respectively. Additionally, $f_{f}$ is the focal length of the lens, $I_{0\;}$ is the intensity of the incoming light, and $I_{\theta }$ is the intensity of the scattering light as a function of angle θ . The Supplementary Material, section S.2, describes more details about the cross-section calculations.

Table 1 shows the calculated scattering cross-section using Eq. 1 for each nanomaterial. The calculated cross-section of GNRs agrees with values reported previously (Jain et al., 2006; Yang and Dogan, 2014). TiN NPs displays a cross-section roughly one order of magnitude less than GNRs. However, the fabrication of TiN clusters from nanoparticle agglomeration significantly increased the scattering up to one and two orders of magnitude compared to GNRs and TiN NPs, respectively. The cross-section values in Table 1 confirm our approach on the heating rate characterization discussion, where TiN clusters showed values below TiN NPs due to its scattering increment. However, clusters showed significantly higher heating rates than GNRs due to their superior reabsorption from scattering.

**TABLE 1:** Particle volume and scattering cross-section values for all nanomaterials compared to literature.

Nanomaterial	Particle volume (nm^3^)	Cross-section scattering (nm^2^)
GNRs (Jain et al., 2006; Yang and Dogan, 2014)	—	∼1E4
GNRs	4.9E4	1.09633E4
TiN NPs	6.5E4	5.78302E3
TiN Clusters	1.4E7	3.9129E5

The improved scattering on TiN clusters translates to the reduction of temperature gradients inside the solution and consequently a more uniform laser heating. The thermal uniformity for all plasmonic solutions was obtained by measuring the heating rate change along the entire volume of 500 μL. The set of thermocouples was displaced along the whole sample from top to bottom in steps of 635 μm (see Figure 2B). The heating rate along each displacement was obtained by the same procedure described in Section 3.1. Once the temperature reached plateau, the laser was turned off to allow the solution to cool down before starting the next step measurements. In addition, if the solution evaporated, more was added to keep a constant volume of 500 μL.

**FIGURE 5:**
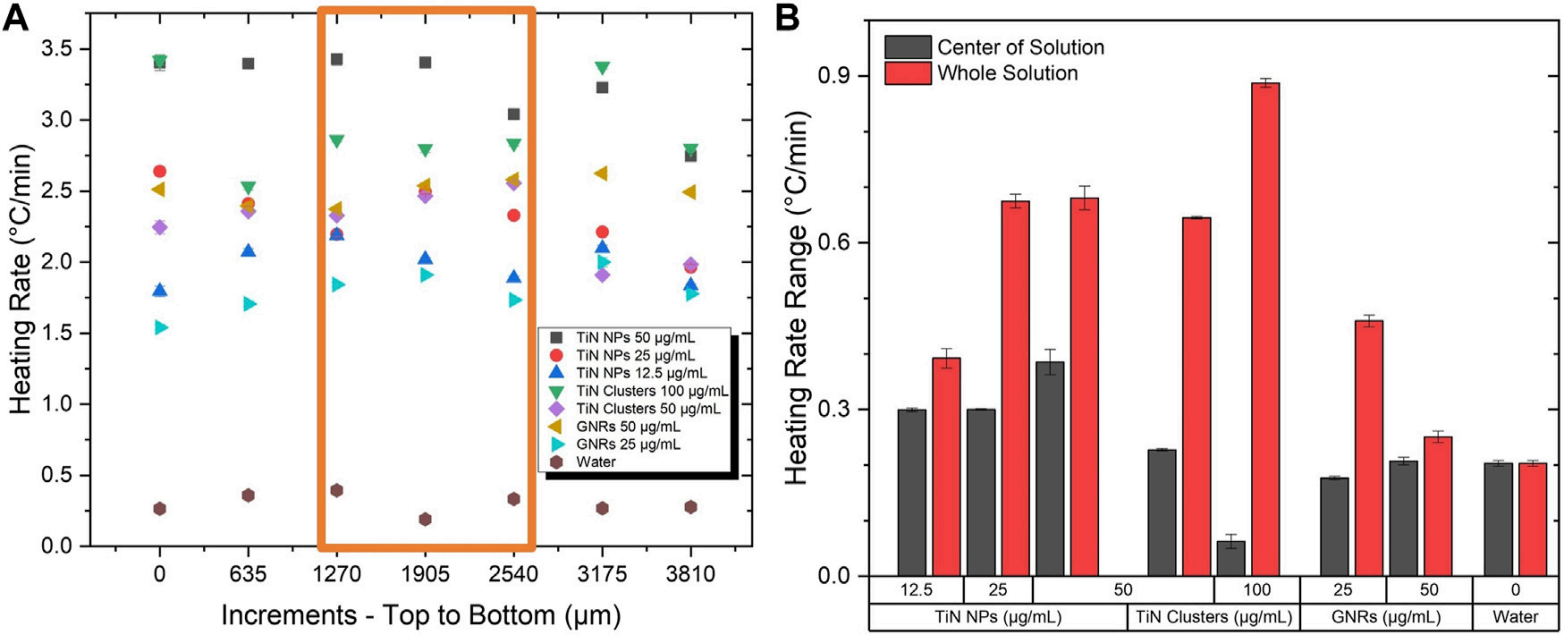
Testing of scattering properties. **(A)** The heating rate at various locations in a volume of 500 μL at a power of 350 mW. **(B)** The change in the heating rate profile in the center where the laser beam passes through and the whole solution.

Figure 5A depicts the heating rate as a function of thermocouple displacement steps. The orange rectangle indicates the area of the laser beam (2 mm) inside the solution. As expected, GNRs showed a more uniform thermal profile along the whole volume compared to TiN NPs due to its higher scattering cross-section. Surprisingly, TiN clusters did not seem to improve uniformity despite its superior scattering cross-section. However, observing only on the laser beam region, the heat uniformity remained almost constant similar to GNRs.

The heating rate change calculations along the whole solution and only on the laser beam area quantified the level of uniformity. For all nanomaterials, Figure 5B demonstrates a higher change in heating rate in the whole solution compared to the laser area, which significantly minimizes. Regardless of the nanomaterial characteristics, the uniformity outside of the laser area is expected to decrease. However, this is more evident for TiN nanomaterials compared to GNRs. Moreover, throughout the laser beam region, the TiN clusters present a similar uniformity to GNRs, even superior at high concentrations (100 μg/mL) with a heating rate change of only 0.1 °C/min. As previously demonstrated, the scattering of TiN clusters is superior to TiN NPs and GNRs (see Table 1). However, at high concentrations, the absorption and reabsorption after scattering of TiN clusters still plays a significant role in the heating process and consequently the thermal uniformity mainly improves in the laser beam spot volume. Importantly, the laser spot and the vitrified microdroplet are the same size in this rewarming application (Khosla et al., 2018). Therefore, this result proposes uniform heating rates throughout rewarming with TiN clusters.

Laser nanowarming requires an optimal ratio between absorption and scattering of the nanomaterials to obtain uniform warming. As this ratio increases, uniform warming is achieved (Liu et al., 2020). This study demonstrated the tunability of scattering for TiN nanomaterials by agglomeration. However, the absorption decreased as the scattering cross-section enhanced with particle size increment. The heating rate of the studied solutions directly relates to its capacity to absorb light, while scattering controls the temperature distribution. While this study does not claim optimization of the absorption-to-scattering ratio, as this likely depends on the specific characteristics of the cryopreserved biomaterial, it shows that controlled agglomeration of TiN nanoparticles provides a pathway towards tuning of its optical properties.

### 3.3 Stability

**FIGURE 6:**
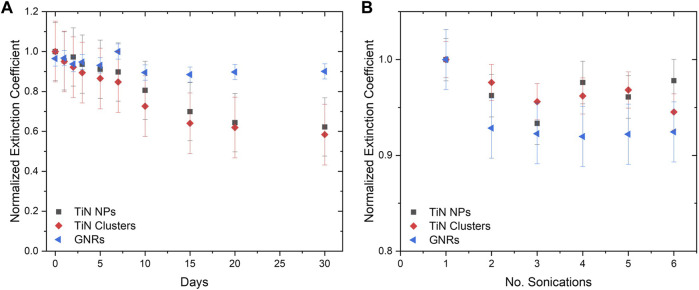
Nanomaterial colloidal stability determined by extinction coefficient. **(A)** The extinction coefficient for 30 days demonstrating the stability of TiN NPs and clusters in comparison to GNRs. **(B)** The extinction coefficient after a certain amount of sonication’s.

The normalized extinction coefficient at the absorption plasmonic peak (λ = 800 nm) over 30 days for TiN NPs, TiN clusters, and GNRs is shown in Figure 6A. Within the first 7 days, TiN NPs and clusters presented a similar extinction coefficient decrease to GNRs of about 10%. This result signifies the optical properties of the CPA plasmonic solutions will remain stable during laser rewarming experiments. The extinction coefficient for TiN NPs and clusters diminished up to 40% after the 30 days, while GNRs only diminished 10%. The GNRs are functionalized with a PEG surface polymer, therefore, they displayed greater colloidal stability. Functionalization in TiN nanomaterials, to increase their stability, requires further exploration in a future study.

Additionally, the colloidal stability of solutions after several rounds of sonication was measured. Figure 6B demonstrates the extinction coefficient of GNRs decreased up to 8%, while TiN clusters and TiN NPs decreased up to 5% and 3% respectively, after six repetitions. The possibility of re-sonication of samples without compromising optical properties is important for the future distribution of these solutions to collaborators for further investigations.

### 3.4 TiN toxicity

**FIGURE 7:**
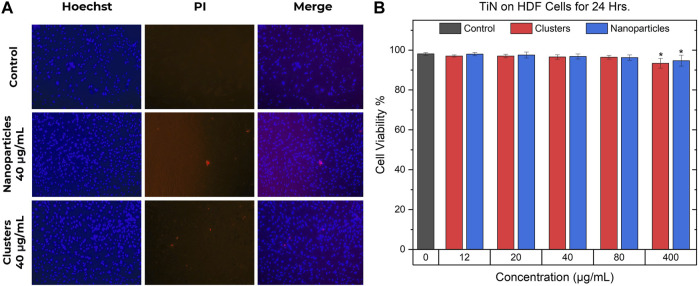
TiN nanoparticles and clusters cell viability. **(A)** Hoechst and PI double staining in HDF cells. The images in the left column show the cells stained with Hoechst, the middle column images show the cells with positive staining of PI, and the right column shows the merged images. The first row shows a control group of cells, the second shows a concentration of 40 μg/mL of TiN NPs treatment, and the last row shows a concentration of 40 μg/mL of TiN clusters treatment. **(B)** Cell viability of HDF cells after 24 h of incubation while being treated with TiN NPs and TiN clusters. (*p* < 0.05*).

Figure 7 shows the results of the toxicity experiments for TiN NPs and TiN clusters carried out in HDF cells. As seen in Figure 7A, the blue images display the Hoechst dye staining all the cells, while the red PI dye shows the dead cells. The merged images, using ImageJ, display the live/dead cells simultaneously. A control and a concentration of 40 μg/mL for both TiN NPs and clusters are depicted in Figure 7A. Subtracting the dead cells from all of them gave the number of viable cells. The cell viability was calculated by dividing the viable cells by all cell count:

$$Cell\;Viability\%=\;\frac{Viable\;Cells}{Viable\;Cells+Dead\;Cells}x100$$ (2)

The cell viability percentages are demonstrated in Figure 7B as mean ± standard deviation from four to six experiments. The statistical significance was analyzed by one-way analysis of variance (ANOVA) or Kruskal-Wallis test and the difference was considered significant if *p* < 0.05*. Only HDF cells treated with TiN clusters and NPs at 400 μg/mL demonstrated a significant difference compared to the control, untreated HDF cells, and other treated groups. Concentrations from 12 to 80 μg/mL for both TiN nanomaterials presented a mean cell viability above 96%. The concentration of 400 μg/mL for TiN clusters and NPs presented a mean cell viability above 93% and 94%, respectively.

### 3.5 Laser rewarming on cryoprotective agent-plasmonic microdroplets

**FIGURE 8:**
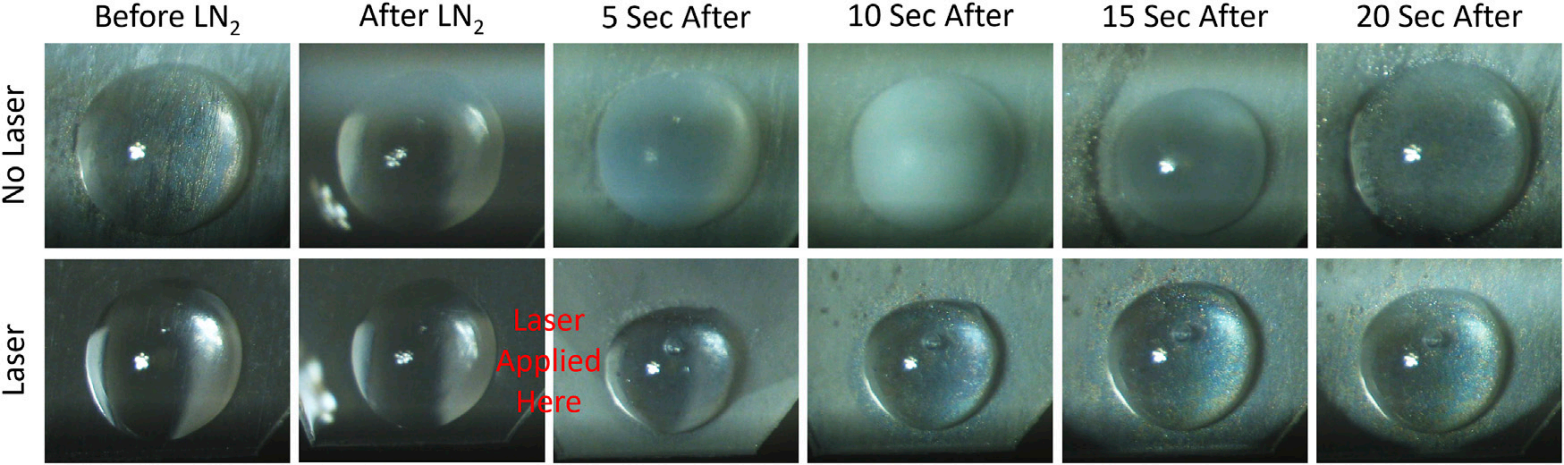
Microdroplet laser rewarming of TiN NPs in CPA solution. The top row displays rewarming a vitrified microdroplet (1 μL) without laser. Devitrification, ice formation, appears as a cloudy white color. The bottom row shows a vitrified microdroplet with laser rewarming and no devitrification.

The laser rewarming process of a vitrified 1 μL droplet is shown on the image sequences of Figure 8. As expected, the cryopreserved droplet that warms up without the assistance of the laser goes through an ice formation stage (top image sequence, white color), which compromises the viability of the biomaterial. Alternatively, the droplet containing TiN NPs at a concentration of 100 μg/mL and heated up by the *ms* pulsed laser presented a successful rewarming, where no ice formation or cracking appeared during the entire process (bottom image sequence). Future work entails measuring the post warming viability of TiN NPs and clusters in actual cryopreserved biomaterials, such as HDF cells and aquatic embryos.

## 4 Conclusion and outlook

Cryopreserved biomaterials at different scales require a precise cooling and warming rate. These rates increase as the scale of the biomaterial decreases and warming at the microliter scale requires assistance from photothermal nanomaterials. TiN nanomaterials are proposed as an alternative to the commonly used GNRs to assist with laser rewarming at this scale. Our study demonstrated the advantages of TiN NPs and TiN clusters, over commercial GNRs. TiN NPs and clusters showed higher temperatures and heating rates compared to GNRs, a promising result for future applications. Uniformity plays another key factor in rewarming to avoid thermal gradients that lead to damage in biomaterials. The scattering of TiN NPs improved by the creation of clusters, which presented a much higher scattering cross-section than both TiN NPs and GNRs, observed by the heating rate change along the volume of the solutions. The uniformity of the thermal profile in the laser spot volume of TiN clusters solutions compared to that of GNRs. In addition, a multiple beam system (4LB) was developed to improve the energy distribution into the sample and therefore improved heating uniformity. The results concluded to an increment of the temperature over time and a heating rate above 50%, in comparison with using only 1LB.

These TiN nanomaterials stand as an alternative to GNRs for warming cryopreserved biomaterials. The colloidal stability of all nanomaterials in CPA solutions was examined *via* extinction coefficient at λ = 800 nm. GNRs and TiN nanomaterials demonstrated a stability of 90% over the first 7 days, but TiN nanomaterials dropped to a value of 60% over a period of 30 days. Also, TiN NPs and clusters demonstrated a stability of 95% after a repetition of sonication of 6 times. These results propose that the optical properties of the CPA plasmonic solution will remain stable during a nanowarming time scale with a possible re-sonication process without damaging the nanomaterials. Additionally, both TiN NPs and clusters, in concentrations ranging from 12 to 80 μg/mL, presented a cell viability over 96% on HDF cells when exposed for 24 h. Furthermore, successful rewarming of a vitrified 1 μL microdroplet consisting of 100 μg/mL TiN NPs in CPA solution was performed using a *ms* 1,064 nm pulsed laser, without visible cracking or ice formation.

In conclusion, TiN NPs and clusters demonstrate higher heating rates and improved heating uniformity over GNRs which makes them suited candidates to use on uniform laser rewarming of cryopreserved biomaterials. Future work entails scaling down our thermometry setup to study and measure the heating rates at μL droplet volumes with pulsed laser radiation and TiN nanomaterials in cryopreserved cells, fish embryos, etc. In addition, further toxicity experiments of TiN nanomaterials in biological samples needs addressing. Moreover, future studies need to address the cryopreservation efficiency and biometrical analysis of specimens with TiN nanomaterials. An in-depth study to address any biological, morphological, or genetic modifications caused by TiN requires further investigation, as well as membrane stability index and genetic analysis to understand the cooling and heating effects in the biomaterials. However, these studies are out of the scope for this work.

## Data Availability

The original contributions presented in the study are included in the article/Supplementary Material, further inquiries can be directed to the corresponding authors.
