# Supplementary material for: Photothermal heating of titanium nitride nanomaterials for fast and uniform laser warming of cryopreserved biomaterials
Source: Front Bioeng Biotechnol. 2022 Aug 25;10:957481. doi: 10.3389/fbioe.2022.957481 (PMC9455577; doi:10.3389/fbioe.2022.957481)
Supplement: Supplementary file 1 [file DataSheet1.PDF]

Supplementary Material

1 Laser Setup

Supplementary Figure 1A shows the one laser beam (1LB) optical setup which consists of two plano-convex lenses with focal lengths of 100 mm (LA1509) and 35.0 mm (LA1027), respectively. A collimated laser (λ = 808 nm), with a spot size of 2 mm, illuminated the plasmonic solution for the photothermal thermometry measurements.

Supplementary Figure 1B shows the four laser beams (4LB) optical system which consisted of three 50:50 non-polarizing beam splitting cubes (BS011) and four broadband dielectric mirrors (BB1-E03) to equally distribute the laser energy into the plasmonic solution. For both setups, an adjustable fiber collimator (CFC5-B) was connected to the output laser to tune the spot diameter. In both setups, a z-micro stage displaced a set of 4 K-type thermocouples along a quartz cuvette containing the plasmonic solutions. The thermocouples were connected to a thermometer reader (Perfect Prime data logger).

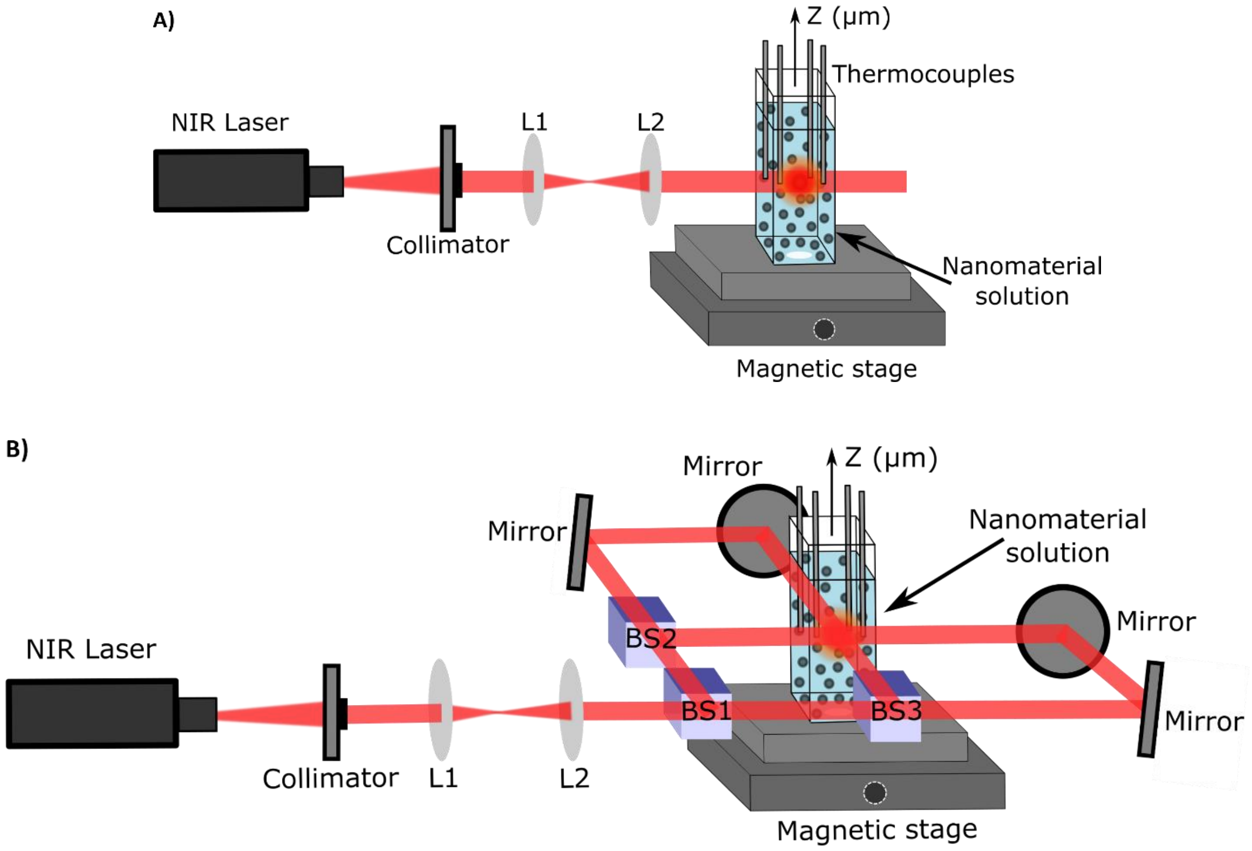

Supplementary Figure 1. Laser setups using CrystaLaser 808 nm laser. (A) Four thermocouple setup for thermal measurements with only 1LB. **(B)** Beam splitting laser setup to uniformly heat solutions from four directions (4LB).

2 Scattering Cross-section

The scattering properties of each nanomaterial were analyzed by measuring the scattering cross-section via multiangle light scattering (MALS) technique. The *CrystaLaser* 808 nm laser was focused into a Pyrex tube, containing the plasmonic solution, using a fiber collimator and lens with a focal length of 75 mm (LA1608), as shown in Supplementary Figure 2A. The scattering light from the solution was collected and focused to an integrating sphere with a lens of f = 50.0 mm (LB1844-B-ML). A calibrated photodetector (DAT01, Thorlabs), attached to the integrating sphere, was connected to an Oscilloscope (Tektronix TDS5104 Digital Phosphor) to measure the scattering signal from the solution. To obtain the scattering intensity at different angles (from 0 to 90°), the integrating sphere was placed on a Thorlabs manual rotating stage (RP01). The power of the laser was set to 350 mW.

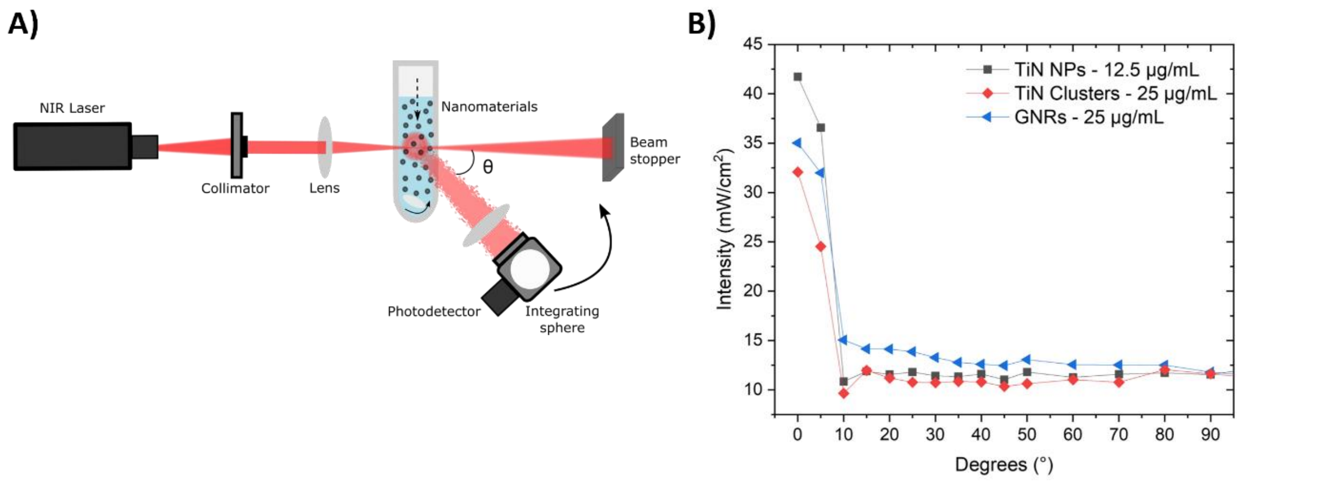

Supplementary Figure 2. Scattering properties. (A) Optical setup to measure scattering cross-section of nanomaterials using a source of 808 nm. The scattering was measured using a photodetector attached to an integrating sphere. **(B)** The intensity measured from scattering signal of TiN NPs, TiN clusters, and GNRs at various angles from 0 to 90 degrees.

Supplementary Figure 2B shows the scattering intensity as a function of angle detection for TiN NPs, TiN clusters, and GNRs, all at their lowest concentrations. The total energy scattered is obtained from:

$$A = \int_{0}^{90} I_{\theta} sin\theta \, d\theta \qquad (1)$$

Where I_{θ} is the intensity of the scattering light as function of angle θ. The scattering cross-section is obtained by:

$$\sigma = \frac{V_{Particle}}{V_{Spot}} \frac{{f_f}^2}{I_{O,Water}} \mathrm{A} \qquad (2)$$

Where $V_{Particle}$ and V_{Spot} are the volume of the nanomaterial and the volume of laser spot, respectively,

f_f is focal length of the lens focusing the light into the integration sphere, and $I_{O,Water}$ is light intensity of only water. Supplementary Table 1 shows the rest of the equations to obtain $V_{Particle}$ and V_{Spot}.

Supplementary Table 1. Equations to Calculate Scattering Cross-Section.

Particle Volume, Sphere	$V_p = \frac{4}{3}\pi r^3$	(3)
Particle Volume, Cylinder	$V_p = \pi r^2 h$	(4)
Beam Waist After Lens	$W_o{}' = \frac{f\lambda}{\pi w_o} x1.5$	(5)
Rayleigh Range	$Z_r = \frac{\pi w_o{}'^2}{\lambda}$	(6)
Spot Volume	$V_s = \pi r^2 h = \pi w_o{}'^2 (2Z_r)$	(7)
